# Comparative speed of kill of sarolaner (Simparica^™^) and afoxolaner (NexGard^®^) against induced infestations of *Ixodes scapularis* on dogs

**DOI:** 10.1186/s13071-016-1307-x

**Published:** 2016-02-15

**Authors:** Robert H. Six, David R. Young, Melanie R. Myers, Sean P. Mahabir

**Affiliations:** Zoetis, Veterinary Medicine Research and Development, 333 Portage St., Kalamazoo, MI 49007 USA; YVRS, 7243 East Ave, Turlock, CA 95380 USA

**Keywords:** *Ixodes scapularis*, Tick, Dog, Sarolaner, Simparica^™^, Afoxolaner, NexGard^®^, Isoxazoline, Oral, Speed of kill

## Abstract

**Background:**

The black-legged (or deer) tick*, Ixodes scapularis*, commonly infests dogs and cats in North America and is the main vector for the pathogen that causes Lyme disease in dogs and humans. The speed of kill of a parasiticide is critical to minimize the direct and deleterious effects of tick infestation and especially to reduce the risk of tick-borne pathogen transmission. In this study, speed of kill of a novel orally administered isoxazoline parasiticide, sarolaner chewable tablets (Simparica^™^), against *I. scapularis* on dogs was evaluated and compared with afoxolaner (NexGard^®^) for five weeks after a single oral dose.

**Methods:**

Twenty four dogs were randomly allocated to treatment with either placebo, sarolaner (2 to 4 mg/kg), or afoxolaner (2.5 to 6.8 mg/kg) based on pretreatment tick counts. Dogs were examined and live ticks counted at 8, 12, and 24 h after treatment and subsequent re-infestations on Days 7, 14, 21, 28 and 35. Efficacy was determined at each time point relative to counts for placebo dogs.

**Results:**

A single oral dose of sarolaner provided >99 % efficacy within 24 h of treatment and >95 % against subsequent weekly re-infestations of ticks consistently to Day 35. For the earlier time points, sarolaner significantly reduced tick counts versus placebo from Day 0 to Day 21 at 8 and 12 h, and on Day 35 at 12 h (*P* ≤ 0.0174), while afoxolaner was only significantly lower at 8 h on Days 0 and 14 (*P* ≤ 0.0309), and at 12 h on Day 0 only (*P* < 0.0001). Significantly more live ticks were recovered from afoxolaner-treated dogs than from sarolaner-treated dogs at 24 h after infestation from Day 14 to Day 35 (*P* ≤ 0.0278). At 24 h, efficacy (based on geometric mean counts) of afoxolaner declined to less than 80 % from Day 21 through the end of the study, while efficacy for sarolaner was >95 % for 35 days. There were no adverse reactions to treatments.

**Conclusions:**

In this controlled laboratory evaluation, sarolaner had a faster speed of kill against *I. scapularis* than afoxolaner. This was noticeably more pronounced towards the end of the monthly treatment period. The rapid and consistent kill of ticks provided by sarolaner within 24 h after a single oral dose and re-infestation over 35 days suggests this treatment will provide highly effective and reliable control of ticks over the entire treatment interval, and should reduce the risk of tick-borne diseases, including Lyme disease whose agent is vectored by *I. scapularis*.

## Background

The black-legged (or deer tick)*, Ixodes scapularis*, is one of the ticks most commonly infesting dogs and cats in North America [1]. The tick species is widely distributed in the northeastern and upper midwestern United States and southeastern to southcentral Canada. The occurence and geographic distribution of *I. scapularis* ticks is increasing due to the mobility of people and pets, as well as the increase in wildlife populations in urban environments. The infestation of migratory birds also contributes to the geographic expansion increasing the disease risk for humans, wildlife, and domestic animals [2, 3].

*Ixodes scapularis* ticks have become an increasingly important concern for public and veterinary health [4]. These ticks serve as vectors of *Borrelia burgdorferi* and *Anaplasma phagocytophilum*, the causative agents of Lyme borreliosis and granulocytic anaplasmosis in humans and dogs, and may also transmit *Babesia microti*. [5, 6]. *Ixodes scapularis* can also secrete a salivary neurotoxin that may cause tick paralysis in dogs and humans [1]. The toxin affects the lower motor neurons of the spinal cord and cranial nerves and produces a progressive, ascending flaccid paralysis [7].

Tick control and prevention have taken on a new importance as awareness of and exposure to tick-borne diseases increases and *I. scapularis* (and other tick species) populations continue to expand. Until recently, topically administered parasiticides with contact activity have been the most common approach to tick control on the dog. A new class of compounds, the isoxazolines, have efficacy against fleas and ticks for 1 month or longer following a single oral dose [8, 9]. These systemically active compounds act rapidly to impact the tick’s feeding behavior and cause death of the ticks. Afoxolaner has been reported to provide >90 % efficacy against *Ixodes ricinus* within 24 h for up to 28 days after a single dose [10], and sarolaner has similarly demonstrated >90 % efficacy against both *I. scapularis* and *I. ricinus* for 28 days [11]. While tick efficacy claims are generally based on evaluation at 48 h after treatment or re-infestation [12], the speed of acaricidal activity is critical in disrupting or preventing feeding and thus reducing the risk of pathogen transmission which generally occurs after the infected tick is attached and feeding for 24 to 48 h [13, 14].

Sarolaner is a novel isoxazoline which in a chewable tablet formulation (Simparica™) provides excellent control of fleas and ticks for at least one month after a single oral dose (TL McTier, personal communications). A laboratory study was conducted to evaluate and compare the speed of kill of sarolaner and afoxolaner (Nexgard^®^) against existing *I. scapularis* infestations and weekly re-infestations for a period of 5 weeks after treatment with a single dose.

## Methods

The study was a masked, negative-controlled, randomized laboratory efficacy design conducted in California, USA. Study procedures were in accordance with the World Association for the Advancement of Veterinary Parasitology (WAAVP) guidelines for evaluating the efficacy of parasiticides for the treatment, prevention and control of flea and tick infestation on dogs and cats [12] and complied with the principles of Good Clinical Practices [15]. The protocol was reviewed and approved by the local Institutional Animal Care and Use Committee. Masking of the study was assured through the separation of functions. All personnel conducting observations or animal care or performing infestations and counts were masked to treatment allocation.

### Animals

Twenty-four, male and female, purpose-bred Beagles from 1 to 3 years of age and weighing from 8.1 to 13.8 kg were used in the study. Each dog was individually identified by a unique ear tattoo or electronic transponder and had undergone an adequate wash-out period to ensure that no residual ectoparasiticide efficacy remained from any previously administered treatments. Dogs were individually housed in indoor runs such that no physical contact was possible between them and were acclimatized to these conditions for at least 14 days prior to treatment. Dogs were fed an appropriate maintenance ration of a commercial dry canine feed for the duration of the study. Water was available *ad libitum*. All dogs were given a physical exam to ensure that they were in good health at enrollment and suitable for inclusion in the study. General health observations were performed twice daily throughout the study.

### Design

The study followed a randomized complete block design. Dogs were ranked according to decreasing tick counts into blocks of three and within each block a dog was randomly allocated to treatment with placebo, sarolaner, or afoxolaner. There were eight dogs per treatment group. Dogs were infested with ticks two days prior to treatment and then weekly for five weeks. Tick counts were conducted at 8, 12, and 24 h after treatment and each subsequent weekly re-infestation.

### Treatment

Bodyweights collected on Day −2 were used to determine the appropriate dose to be administered. On Day 0, dogs received either a placebo tablet, the appropriate strength sarolaner chewable tablet (Simparica^™^), to provide sarolaner at the recommended dose of 2 mg/kg (range: 2 to 4 mg/kg), or NexGard^®^ per label directions (afoxolaner at 2.5 to 6.8 mg/kg). All doses were administered by hand pilling to ensure accurate and complete dosing. Each dog was observed for several minutes after dosing for evidence that the dose was swallowed, and for general health at 1, 4, and 24 h after treatment administration.

### Tick infestation and assessment

The ticks were obtained from the Oklahoma State University’s *I. scapularis* colony which was initiated in 1991 with engorged females collected locally in Stillwater, OK. The colony has been maintained with the introduction of engorged females collected from the local natural population every two years.

Tick infestations were performed on Days −7 (host suitability and allocation), −2, 7, 14, 21, 28, and 35. Prior to each infestation, the dog was lightly sedated with ketamine/xylazine and a precounted aliquot of 50 (±5) viable unfed adult *I. scapularis* were directly applied to the dog, which was then confined in an appropriately sized travel crate for 4 h to restrict movement and facilitate tick attachment. Each dog was examined to remove and count live ticks at 48 h after the initial host suitability infestation. At 8 and 12 (±0.5) hours after treatment and each subsequent weekly re-infestation, the dogs were examined systematically so that the entire body surface was carefully examined and live ticks were counted *in situ*. At 24 h after treatment and each subsequent weekly re-infestation, the dogs were examined and then thoroughly combed to count and remove live ticks. Each dog was examined for at least 10 min. If ticks were encountered in the last minute, combing was continued in 1 min increments until no ticks were encountered.

### Statistical analysis

The individual dog was the experimental unit and the primary endpoint was live tick counts. Data for post-treatment live (free plus attached) tick counts were summarized with arithmetic (AM) and geometric (GM) means by treatment group and time point. Tick counts were transformed by the log_e_(count + 1) transformation prior to analysis in order to stabilize the variance and normalize the data. Using the PROC MIXED procedure (SAS 9.2, Cary NC), transformed counts were analyzed using a mixed linear model. The fixed effects were treatment, timepoint and the interaction between timepoint and treatment by timepoint. The random effects included block, block by treatment interaction, and error. Testing was two-sided at the significance level α = 0.05.

The assessment of efficacy for live ticks was based on the percent reduction in the AM and GM live tick counts relative to placebo and to the positive control, as suggested by the most recent guidelines of the WAAVP for systemic acaricides [11], and was calculated using Abbott’s formula:

$$ \%\ \mathrm{reduction}=100 \times \frac{\mathrm{mean}\ \mathrm{count}\ \left(\mathrm{placebo}\right)\hbox{--} \mathrm{mean}\ \mathrm{count}\ \left(\mathrm{treated}\right)}{\mathrm{mean}\ \mathrm{count}\ \left(\mathrm{placebo}\right)} $$

## Results

There were no treatment-related adverse events during the study. Placebo-treated dogs maintained good tick infestations throughout the study with mean tick counts ranging from approximately 20 to 33 (Tables 1, 2 and 3).

**Table 1:** Mean live *Ixodes scapularis* counts and efficacy relative to placebo at 8 h after treatment and post-treatment re-infestations for dogs treated with a single oral dose of sarolaner or afoxolaner on Day 0

Treatment		Day of treatment or re-infestation					
		0	7	14	21	28	35
Placebo	Range	20–40	12–32	17–37	18–40	12–32	15–34
	A. mean	30.3	25.5	25.4	28.6	24.1	23.8
	G. mean^1^	29.4^a^	24.2^a^	24.5^a^	27.8^a^	23.2^a^	23.0^a^
Sarolaner	Range	15–25	11–24	10–30	13–36	16–35	13–36
	A. mean	20.8	16.8	16.1	19.6	25.6	23.1
	Efficacy (%)	31.4	34.3	36.5	31.4	0.0	2.6
	G. mean^1^	20.5^b^	16.2^b^	15.2^b^	18.5^b^	25.0^a^	21.9^a^
	Efficacy (%)	30.3	32.8	38.0	33.5	0.0	5.0
	*P*-value vs. placebo	0.0082	0.0174	0.0070	0.0065	0.5822	0.6940
Afoxolaner	Range	12–27	12–27	11–26	17–36	15–34	17–25
	A. mean	18.4	21.8	17.6	23.3	23.6	20.0
	Efficacy (%)	39.3	14.7	30.5	18.8	2.1	15.8
	G. mean^1^	17.8^b^	21.1^a,b^	17.0^b^	22.7^a,b^	22.7^a^	19.9^a^
	Efficacy (%)	39.6	12.5	30.8	18.5	1.9	13.8
	*P*-value vs. placebo	0.0006	0.3909	0.0309	0.1445	0.8889	0.2619
	*P*-value vs. sarolaner	0.2569	0.1028	0.5023	0.1487	0.4914	0.4585

^1^Geometric means within columns with the same superscript are not significantly different (*P* > 0.05)

**Table 2:** Mean live *Ixodes scapularis* counts and efficacy relative to placebo at 12 h after treatment and post-treatment re-infestations for dogs treated with a single oral dose of sarolaner or afoxolaner on Day 0

Treatment		Day of treatment or re-infestation					
		0	7	14	21	28	35
Placebo	Range	20–43	13–30	10–39	15–36	15–36	18–33
	A. mean	32.9	23.1	24.4	26.3	23.9	25.4
	G. mean^1^	32.0^a^	22.3^a^	22.6^a^	25.4^a^	23.1^a^	24.7^a^
Sarolaner	Range	4–22	6–14	0–18	1–26	12–35	1–29
	A. mean	9.1	9.5	12.1	10.3	18.6	15.4
	Efficacy (%)	72.2	58.9	50.3	61.0	22.0	39.4
	G. mean^1^	8.0^b^	9.1^b^	9.5^b^	7.9^b^	17.5^a^	11.6^b^
	Efficacy (%)	74.9	59.2	58.0	68.8	24.0	53.3
	*P*-value vs. placebo	<0.0001	<0.0001	0.0120	0.0002	0.1054	0.0131
Afoxolaner	Range	5–15	11–29	12–21	12–40	11–38	14–23
	A. mean	10.6	18.5	15.9	23.6	21.5	19.3
	Efficacy (%)	67.7	20.0	34.9	10.0	9.9	24.1
	G. mean^1^	10.2^b^	17.6^a^	15.6^a,b^	22.3^a^	20.1^a,b^	19.0^a,b^
	Efficacy (%)	68.1	21.2	31.1	12.0	13.0	23.3
	*P*-value vs. placebo	<0.0001	0.1335	0.2460	0.6168	0.4013	0.3512
	*P*-value vs. sarolaner	0.3063	0.0004	0.1369	0.0006	0.4123	0.0949

^1^Geometric means within columns with the same superscript are not significantly different (*P* > 0.05)

**Table 3:** Mean live *Ixodes scapularis* counts and efficacy relative to placebo at 24 h after treatment and post-treatment re-infestations for dogs treated with a single oral dose of sarolaner or afoxolaner on Day 0

Treatment		Day of treatment or re-infestation					
		0	7	14	21	28	35
Placebo	Range	26–39	3–32	12–32	4–36	16–32	14–29
	A. mean	32.4	23.9	23.4	27.5	23.5	22.9
	G. mean^1^	32.0^a^	20.5^a^	22.4^a^	23.9^a^	23.1^a^	22.4^a^
Sarolaner	Range	0–1	0–1	0–0	0–3	0–5	0–17
	A. mean	0.1	0.3	0.0	0.5	1.3	2.6
	Efficacy (%)	99.6	99.0	100	98.2	94.7	88.5
	G. mean^1^	0.1^b^	0.2^b^	0.0^c^	0.3^c^	0.8^c^	1.0^c^
	Efficacy (%)	99.7	99.1	100	98.8	96.6	95.7
	*P*-value vs. placebo	<0.0001	<0.0001	<0.0001	<0.0001	<0.0001	<0.0001
Afoxolaner	Range	0–2	0–6	0–9	1–15	1–21	0–25
	A. mean	0.3	1.8	2.4	6.9	9.0	12.1
	Efficacy (%)	99.2	92.7	89.8	75.0	61.7	47.0
	G. mean^1^	0.1^b^	1.0^b^	1.1^b^	5.3^b^	6.5^b^	6.8^b^
	Efficacy (%)	99.5	95.1	95.1	77.9	71.8	69.6
	*P*-value vs. placebo	<0.0001	<0.0001	<0.0001	0.0003	0.0009	0.0310
	*P*-value vs. sarolaner	0.7558	0.1197	0.0278	<0.0001	0.0001	0.0083

^1^Geometric means within columns with the same superscript are not significantly different (*P* > 0.05)

At the 8-h timepoint, treatment with sarolaner resulted in significantly lower tick counts than placebo-treated dogs (*P* ≤ 0.0174) from treatment through Day 21, and efficacy (GM) ranged from 30.3 to 38.0 % for this period (Table 1). Treatment with afoxolaner resulted in significantly lower tick counts than placebo at 8 h on Days 0 and 14 only (*P* ≤ 0.0309), with efficacy (GM) of 39.6 and 30.8 %, respectively (Table 1). There were no significant differences between the mean live tick counts at 8 h for the sarolaner- and afoxolaner-treated dogs on any day (*P* ≥ 0.1028).

At the 12-h timepoint, sarolaner-treated dogs had significantly lower tick counts than placebo-treated dogs (*P* ≤ 0.0131) from treatment through Day 21, and on Day 35, with efficacy (GM) ranging from 53.3 to 74.9 % (Table 2). Treatment with afoxolaner resulted in significantly lower tick counts than placebo at 12 h on Day 0 only (*P* < 0.0001), with efficacy (GM) of 68.1 %. Efficacy for afoxolaner was ≤31.1 % on all other days (Table 2). Tick counts were significantly higher for afoxolaner-treated dogs than for sarolaner-treated dogs on Days 7 and 21 (*P* ≤ 0.0006).

**Fig. 1:**
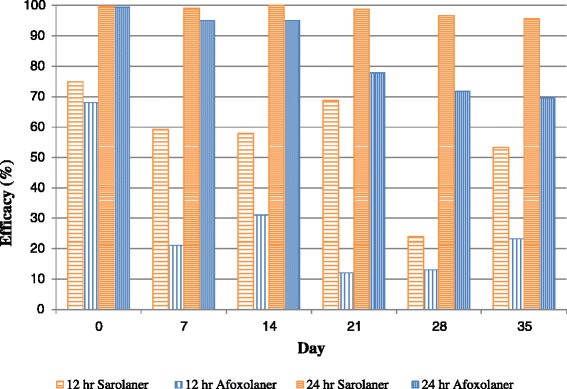
Percent efficacy based on geometric mean counts relative to placebo at 12 and 24 h after treatment and weekly post-treatment re-infestations of *Ixodes scapularis* for dogs treated with a single oral dose of sarolaner or afoxolaner on Day 0

At the 24-h time point, both treatments resulted in significantly lower tick counts than placebo-treated dogs (*P* ≤ 0.0310) throughout the study, and sarolaner-treated dogs also had significantly fewer ticks than afoxolaner-treated dogs (*P* ≤ 0.0278) on Days 14, 21, 28, and 35 (Table 3). Treatment with sarolaner resulted in efficacy (GM) of at least 95.7 % through Day 35, while efficacy for dogs treated with afoxolaner declined below 80 % from Day 21 onwards (Table 3, Fig. 1).

## Discussion

A single dose of sarolaner resulted in the rapid reduction of an existing infestation with *I. scapularis* ticks and the rapid kill of re-infestations for 5 weeks after treatment. Efficacy of >90 % was achieved within 24 h for 28 days. Rapid kill of ticks is key to ameliorating the direct adverse effects of tick feeding and is critical in reducing the risk of transmission of tick-borne pathogens. Transmission of *B. burgforferi* is considered not to occur until ticks have been attached for at least 48 h [16, 17], and transmission rates of *B. burgdorferi* and *A. phagocytophilum* have been shown to drop significantly if ticks are prevented from feeding for more than 24 h [18]. Thus, the rapid efficacy of a single oral dose of sarolaner demonstrated here that resulted in the kill of the majority of ticks within 24 h of weekly re-infestations for 1 month should provide a marked reduction in the risk of a treated dog becoming infected with the pathogens transmitted by *I. scapularis*.

Efficacy of afoxolaner against *I. scapularis* has been described previously with assessment at 48 h after treatment and weekly re-infestations; Mitchell et al. [19] reported efficacy (GM) of 98.4 % against an existing infestation and ≥94.2 % against re-infestations up to Day 28. This level of efficacy for afoxolaner at 48 h is similar to the efficacy achieved by sarolaner at 24 h in this study, and further highlights the speed of tick kill following a single dose of sarolaner. Also notable was the consistency of sarolaner’s efficacy at 24 h after re-infestations over the duration of the treatment interval, while efficacy of afoxolaner at 24 h declined significantly to less than 80 % from Day 21 onwards. This consistent and rapid kill of ticks within 24 h over a full month following a single administration of sarolaner supports the potential for the product to aid in the prevention of the transmission of tick-borne pathogens to dogs.

## Conclusions

This study confirmed the excellent acaricidal efficacy of sarolaner against *I. scapularis* after a single oral administration. Ticks were killed rapidly, with the majority of ticks killed within 24 h after weekly re-infestations for 5 weeks. Efficacy for sarolaner-treated dogs was consistently higher than that of afoxolaner from Day 14 onwards at 24 h. Thus, sarolaner chewable tablets (Simparica^™^) offers the pet owner and the veterinarian an efficacious oral product with a rapid speed of kill of ticks over the entire month following a single oral dose, and will be an important new tool in the treatment and prevention of tick infestation, and should reduce the risk of tick–borne pathogen transmission.
